# Engineered Proteins and Materials Utilizing Residue-Specific Noncanonical Amino Acid Incorporation

**DOI:** 10.1021/acs.chemrev.3c00855

**Published:** 2024-07-15

**Authors:** Temiloluwa Majekodunmi, Dustin Britton, Jin Kim Montclare

**Affiliations:** † Department of Chemical and Biomolecular Engineering, New York University Tandon School of Engineering, Brooklyn, New York 11201, United States; ‡ Department of Biomedical Engineering, New York University Tandon School of Engineering, Brooklyn, New York 11201, United States; § Bernard and Irene Schwartz Center for Biomedical Imaging, Department of Radiology, New York University School of Medicine, New York, New York 10016, United States; ∥ Department of Chemistry, New York University, New York, New York 10012, United States; ⊥ Department of Biomaterials, New York University College of Dentistry, New York, New York 10010, United States; # Department of Radiology, New York University Langone Health, New York, New York 10016, United States

## Abstract

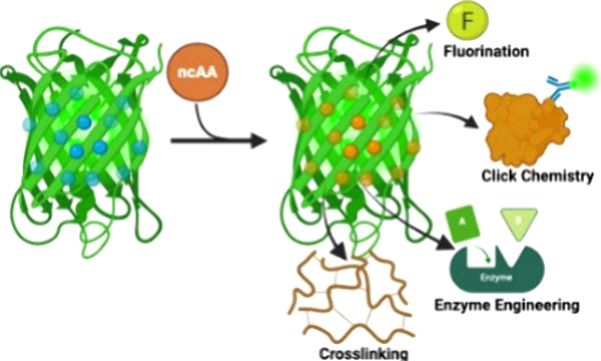

The incorporation of noncanonical amino acids into proteins and protein-based materials has significantly expanded the repertoire of available protein structures and chemistries. Through residue-specific incorporation, protein properties can be globally modified, resulting in the creation of novel proteins and materials with diverse and tailored characteristics. In this review, we highlight recent advancements in residue-specific incorporation techniques as well as the applications of the engineered proteins and materials. Specifically, we discuss their utility in bio-orthogonal noncanonical amino acid tagging (BONCAT), fluorescent noncanonical amino acid tagging (FUNCAT), threonine-derived noncanonical amino acid tagging (THRONCAT), cross-linking, fluorination, and enzyme engineering. This review underscores the importance of noncanonical amino acid incorporation as a tool for the development of tailored protein properties to meet diverse research and industrial needs.

## 1 Introduction

Proteins, with their crucial roles as enzymes, hormones, and neurotransmitters in living systems,^1^ have garnered substantial interest for facilitating various chemical processes. Since the discovery of diastase in the 1800s,^2^ there has been a sustained effort to adapt these versatile compounds for broader utility beyond their inherent biological roles. With the development of recombinant DNA technology and directed evolution techniques, it has become possible to tune proteins toward specific predefined functions, increasing their value.^3,4^ Crucial compounds like antibodies, enzymes, and therapeutics can now be produced efficiently in large quantities.^5^

The applications of engineered proteins are limitless. Especially within the framework of green chemistry, proteins have emerged as ideal facilitators for greener processes, making them essential for addressing current environmental challenges.^6,7^ Owing to their high biocompatibility and biodegradability, protein engineered materials are viewed as viable replacements for the current synthetic polymers.^8^

A key driver behind the popularity of proteins as biomaterials lies in their interchangeable library of amino acids within a sequence.^9^ This has allowed for the development of specific protein functions and further tunability by simple reordering of a sequence from a 20 amino acid library.^10^ With the advent of noncanonical amino acids (ncAAs), the permutations of protein sequences, and subsequently their functions, have diversified enormously.^11,12^

There have been several comprehensive reviews over the years on different aspects of the incorporation of ncAAs into proteins.^13−20^ In this review, we explore the methods and applications of ncAA-bearing proteins and peptide materials, specifically those with ncAAs incorporated through residue-specific means. Residue-specific incorporation offers a unique capability to globally modify protein properties, leading to proteins that usually display substantially altered physical and chemical characteristics.^18^

We cover recent developments for (1) bio-orthogonal noncanonical amino acid tagging (BONCAT), fluorescent noncanonical amino acid tagging (FUNCAT), and threonine-derived noncanonical amino acid tagging (THRONCAT); (2) cross-linking; (3) fluorination; and (4) enzyme engineering. These advancements highlight the pivotal role of residue-specific incorporation in creating novel proteins with tailored functionalities.

## 2 Canonical and Noncanonical Amino Acids

**Figure 1:**
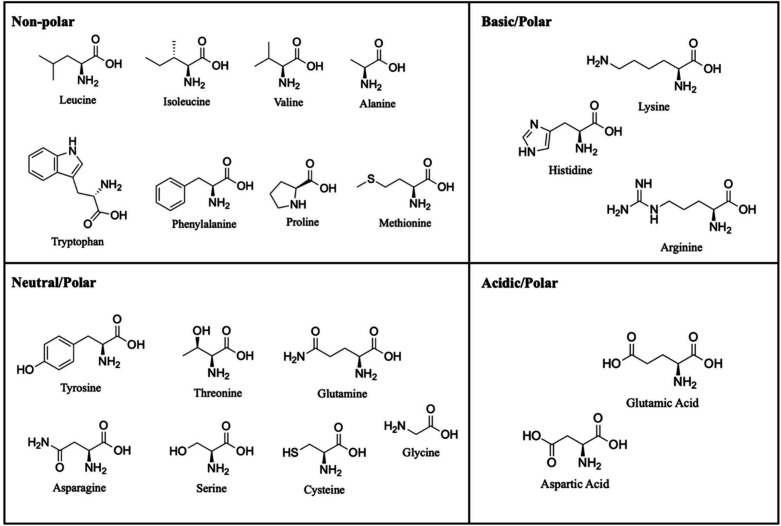
Structures of the 20 canonical amino acids.

Amino acids are the building blocks of proteins, and they allow for endless combinations, forming distinct domains within proteins that govern their structure, function, and stability.^21,22^ The standard genetic code gives rise to 20 amino acids, popularly referred to as the canonical amino acids (cAAs), which are incorporated into proteins biosynthetically during translation. All 20 amino acids share a common backbone (
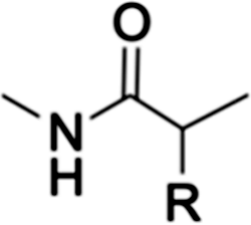
) containing a carboxyl and amino group involved in subsequent protein peptide bonding,^23^ and they can be grouped based on their variable side chains (Figure 1). First and foremost, amino acids can be classified based on polarity (polar and nonpolar), which importantly dictates protein hydrophobicity.^23^ Polar amino acids can be further classified based on charge at neutral pH (pH 7.0).^24^ Histidine (His) is particularly noteworthy, due to its p*K*_a_ being approximately 6.0, resulting in either its protonation or deprotonation at neutral pH depending on the local environment.^25^ Another amino acid of note is cysteine (Cys), which is capable of forming disulfide bonds in oxidative environments and is often found in enzyme active sites due to its nucleophilic behavior.^25^

**Figure 2:**
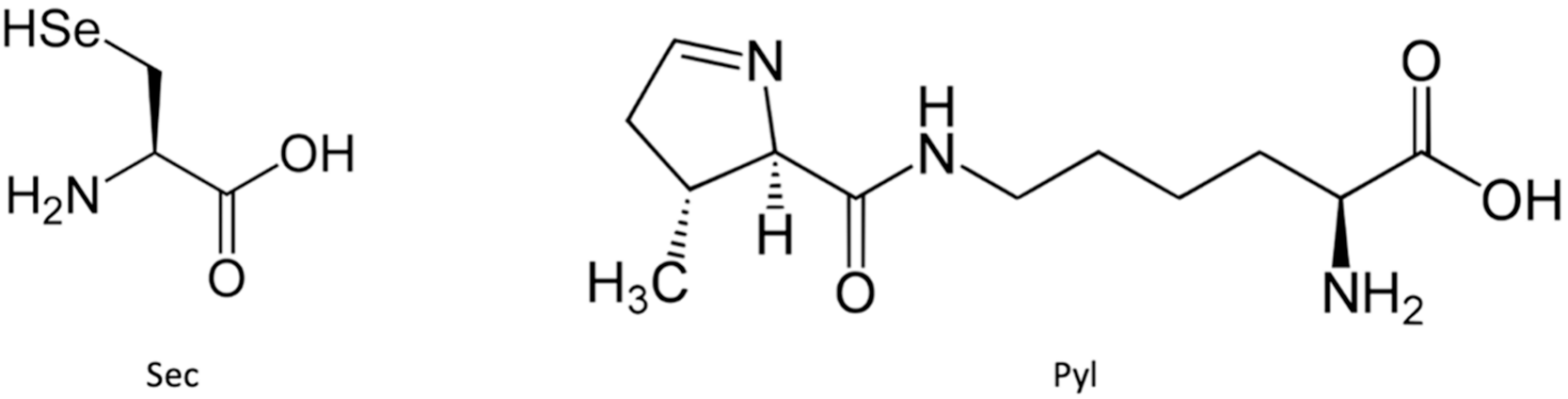
Structures of the other proteinogenic amino acids: selenocysteine (Sec) and pyrrolysine (Pyl).

There are two other proteinogenic amino acids: selenocysteine (Sec) is a Cys analogue with a selenium-containing selenol group in place of the sulfur-containing thiol group^26^ and pyrrolysine (Pyl) is a lysine (Lys) analogue that appears only in proteins of archaea organisms and a few bacterial genera^27^ (Figure 2). Sec is encoded by UGA, which is normally a stop codon but in the presence of the required machinery leads to the cotranslational incorporation of Sec.^28,29^ There are only 25 selenoproteins in the human proteome^30^ so Sec is very rare, although some of those proteins have been shown to be integral for mammalian life.^31,32^ Pyl, a derivative of Lys with an attached pyrroline ring, is similarly encoded by an amber stop codon, UAG.^28^ These amino acids are technically the 21st and 22nd genetically encoded amino acids, though more than traditional translation must occur for their incorporation.

While the cAAs are crucial, their utility is constrained by the limited functional groups and chemistries they offer. Even within the proteome, post-translational modifications (PTMs) must occur to sustain everyday life.^33^ For more scientific and biomedical applications, a diversification of protein structures and chemistries is necessary. As a result, ncAAs have emerged as a significant advancement in the development of functional biomaterials, further enhancing their potential.^34^

As the name implies, ncAAs are amino acids that are not coded for in the genetic code,^21^ and the added diversity of ncAAs suggests a world of possibilities for proteins and protein engineering. They can be derived biologically with engineered bacterial strains or chemically through a variety of pathways as outlined by Adhikari et al.^35^ Notably, asymmetric catalytic synthesis is highlighted as the most efficient and effective way to produce optically enriched α-amino acids.^36−39^

The incorporation of ncAAs can occur either site specifically, where the ncAA is added at a specific site, or residue specifically, which involves globally replacing a cAA with a noncanonical one. Both methods are distinct yet complementary, offering unique advantages depending on their use.^40,41^ The push for ncAAs has been intense, and many ncAA-bearing proteins with improved stability and varied functionalities have been produced.^42−51^

## 3 Methods of ncAA Incorporation

### 3.1 Site-Specific Incorporation (SSI)

**Figure 3:**
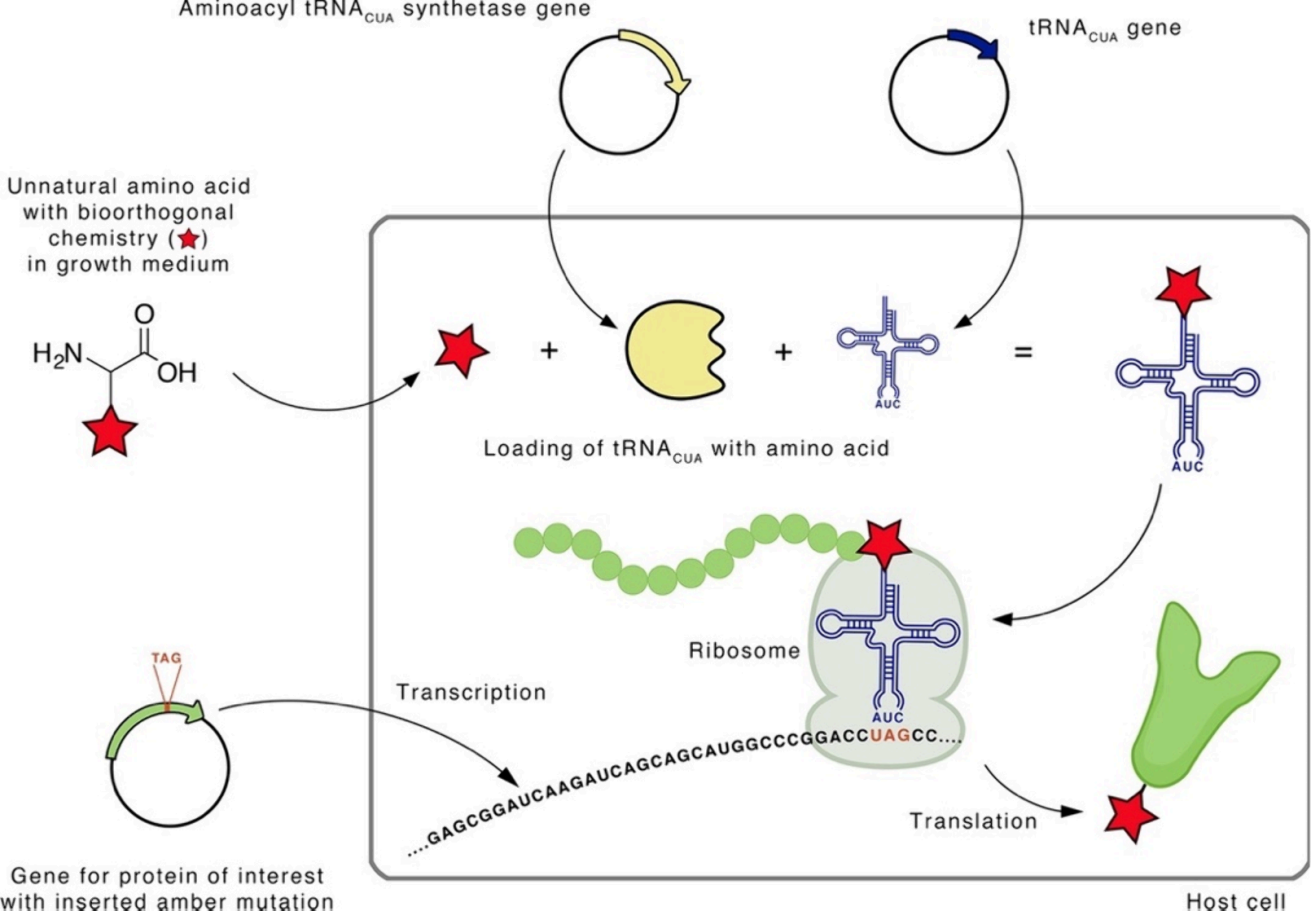
Schematic representation of the site-specific incorporation of ncAAs into proteins by amber suppression. In this method, the gene for the protein of interest is mutated with an amber codon at the desired site of modification. A mutant aminoacyl-tRNA_CUA_ synthetase recognizes the ncAA bearing the desired functionality and loads it to the corresponding tRNA_CUA_. This leads to the expression of the protein of interest with the desired functionality at the genetically encoded site. Reprinted from ref (54). Copyright 2013 American Chemical Society.

Site-specific incorporation (SSI) involves the reassignment of specific codons in the genetic code to an ncAA.^52^ Genetically encoding an ncAA requires a blank codon that can be used to encode the ncAA, a corresponding tRNA that only recognizes this codon, and a new aminoacyl-tRNA synthetase that recognizes the ncAA and loads it to the corresponding tRNA.^53,54^ The most established method is stop codon suppression (SCS), which is based on the fact that only one stop codon is needed for the termination of protein translation, leaving the other two to be reassigned to an ncAA.^55^ The amber codon (UAG) is the preferred choice for recoding as it is used the least in *Escherichia coli* (∼7%) and rarely terminates essential genes.^56^ First, an amber codon is inserted into the desired modification site of the target protein. A suppressor tRNA_CUA_ and a corresponding aminoacyl-tRNA_CUA_ synthetase, which has been evolved to selectively bind the ncAA to the suppressor tRNA_CUA_, are used. The tRNA_CUA_ prevents translation termination, and instead the ncAA is inserted in response to the amber codon (Figure 3). Efforts are ongoing to develop new tRNA synthetase pairs to expand the range of ncAAs that can be incorporated.^57−60^

Another method of SSI of ncAAs is based on quadruplet codons (four nucleotides).^61^ Here, the translational machinery is engineered to allow quadruplet codons to be decoded. These codons are then used as insertion signals for encoding the incorporation of ncAAs into proteins.^62^ The main drawback of suppressor-based methods in general is the relatively limited capacity of suppressor tRNA loaded with ncAAs to fully suppress nonsense (triplet or quadruplet) codons.^63^

One method to overcome this is led by the Romesberg lab in the expansion of the genetic code to allow for direct ncAA incorporation in fully orthogonal base pairings.^64^ Here, codons that consist of an unnatural fifth and sixth nucleotide base pair (UNP) in semisynthetic organisms (SSOs) allow for efficient decoding of many new codons for ncAAs. These UNPs have been generated based on nucleotides that pair through hydrogen bonding,^65^ shape analogues^66^ with a wide variety of functional groups, and *de novo* development of aromatic nucleobase analogues through hydrophobic and packing interactions.^67−69^ While these methods offer great variety, UNPs provide challenges in chemical optimization and can suffer from inefficient DNA replication in some sequences.^64^

Another approach has been to compress the *E. coli* genome codons through removing unnecessary synonymous codons. The Chin lab has reduced the 64-codon genome to 61 codons allowing for an organism to use just 59 sense codons to encode the 20 amino acid library.^70^ The group moreover has investigated the ability to reassign codons encoding for serine, leucine, and alanine and found a variety of successes depending on the target;^71^ they have also investigated the ability to encode multiple ncAAs through orthogonal pyrrolysyl-tRNA synthetase/tRNA pairs.^72^ Schepartz and Söll have demonstrated that these synonymous sense codons can be further reassigned to efficiently translate proteins with ncAAs.^73^

### 3.2 Residue-Specific Incorporation (RSI)

Residue-specific incorporation (RSI) involves the reassignment of sense codons in the *in vivo* expression of recombinant proteins.^74^ By careful engineering, the sense codons of a cAA can be reassigned to a noncanonical one producing a protein that typically contains 19 cAAs and 1 ncAA.^75^ Most recently, the simultaneous incorporation of multiple ncAAs has been performed.^76^

**Figure 4:**
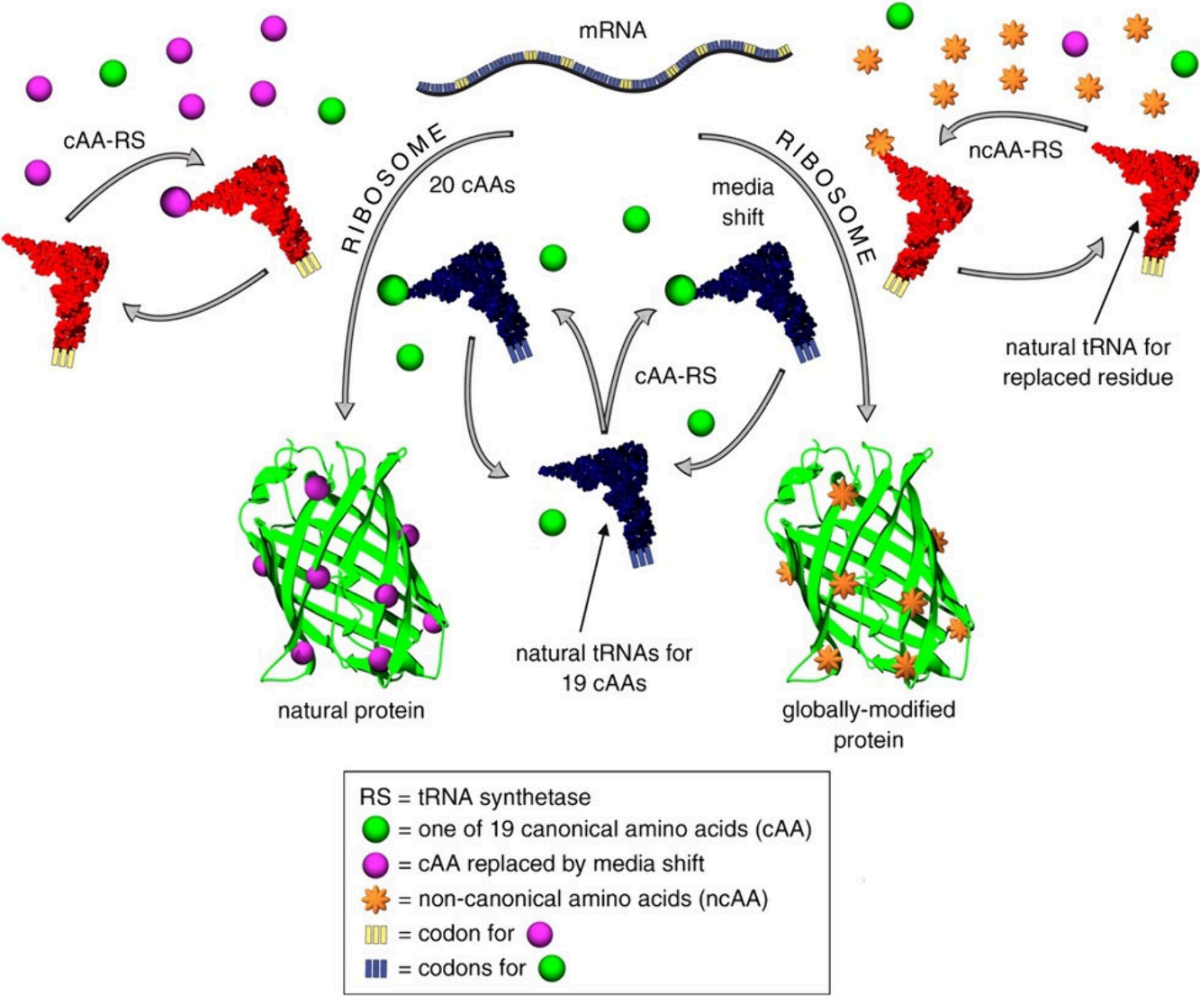
Schematic for selective pressure residue-specific incorporation of ncAAs into proteins. A natural mRNA contains codons for the 20 cAAs. A cAA (purple sphere) assigned to one of those codons (yellow) is replaced with an ncAA (orange star). A medium shift is performed to remove the cAA to be replaced (purple sphere) and to introduce the ncAA (orange star) along with the remaining 19 cAAs (green spheres). The ncAA is charged to the appropriate tRNA (red) by either the wild-type or a mutant aminoacyl-tRNA synthetase (aaRS). The correctly aminoacylated tRNAcAA (blue with green sphere) and the misacylated tRNA (red with orange star) are processed by the ribosome to give a globally modified protein. The left path depicts normal protein synthesis with 20 cAAs for comparison. Reprinted with permission from ref (18). Copyright 2010 Elsevier Ltd.

RSI predominantly relies on the use of an auxotrophic cell line; this allows one to deprive a protein expression medium of a cAA that may then be partially or fully replaced by an ncAA^77^ (Figure 4). An auxotroph is a microorganism that is unable to synthesize one or more essential growth factors and will not grow in media lacking the missing factor. An amino acid auxotrophic expression host is unable to synthesize one of the amino acids so by using chemically defined media; it is possible to control the amino acids available to the host and direct the incorporation of an ncAA.

RSI was first utilized by Cowie and Cohen^78^ where the replacement of Methionine (Met) by its analogue selenomethionine was achieved in an *E. coli* methionine auxotroph. In the simplest iteration of this method, the ncAA must be structurally similar to the canonical one so that the biological machinery of the host accepts it.^15,79^ The engineering of the biological machinery, particularly the aminoacyl-tRNA synthetases (aaRS), has significantly expanded the range of ncAAs that can be incorporated as will be discussed in later sections.

The process typically starts with growing the expression host in a rich medium with all 20 cAAs until there is enough cellular material for protein synthesis. The cells are separated out by centrifugation and go through a cycle of saline washes and centrifugations to completely remove the medium. The cells are then resuspended in a medium with 19 amino acids, without the amino acid that will be replaced and further incubated. This starvation period ensures that the cAA to be replaced is fully consumed, allowing for its preferential replacement by an ncAA in the host cell. The ncAA is then added to the medium in the presence of an inducing agent to initiate protein expression. Some approaches utilize limiting concentrations of the cAA to be replaced to ensure sufficient cell growth and depletion of the cAA, thus obviating the need for salt washes.^76^

The main advantage of this method is its ability to make significant changes in the physical and chemical properties of a protein without any genetic manipulation.^18^ However, it is not conducive for precise control of protein modification,^11^ and depending on the ncAA, it has been shown to have lower incorporation efficiencies than SSI.^19^

#### 3.2.1 Scale-Up Strategies

Obtaining enantiomerically pure ncAAs in the quantities required for large-scale applications continues to pose a challenge, though the emerging biocatalytic production methods are proving to be suitable alternatives in terms of cost and sustainability.^11,35,80,81^ Biocatalytic synthesis also offers the opportunity of coupling the production of the ncAAs with their incorporation into proteins.^80−83^

Schipp et al. have demonstrated this by reconfiguring the methionine biosynthetic pathway in an *E. coli* strain to create a trans-sulfuration pathway for the in-cell production and incorporation of l-azidohomoalanine (Aha). The production rate of Aha sustains cell growth and protein production at a level comparable to cells cultivated in the presence of nonlimiting amounts of methionine.^83^ Similarly, Budisa and colleagues have engineered an *E. coli* strain for the *in vivo* production and incorporation of *S*-allyl-l-homocysteine (Sahc). However, the host is not powerful enough to produce Sahc to levels that would enable efficient competition with residual intracellular methionine resources.^82^ Won et al. have developed a system for the *in vivo* biosynthesis of various tyrosine analogues using phenol derivatives and the tyrosine phenol lyase machinery, and their concurrent incorporation into target proteins by the residue-specific approach.^84^ This method simplifies the incorporation of ncAAs into a single step, eliminating the necessity for a growth and starvation stage.

Ilamaran et al. have also demonstrated a method to streamline the integration of ncAAs by incorporating single ncAAs into different proteins using a single expression host system.^85^ 3, 4-Dihydroxy-l-phenylalanine (DOPA), a tyrosine surrogate that has gained popularity as a metal chelating and detection agent,^41,86,87^ has been incorporated into two proteins, green fluorescent protein (GFP) and annexin V, which are expressed in *E. coli*. This process could substantially reduce the manpower and production costs involved with RSI as different proteins may be used in the same setup.

### 3.3 Cell-Free Protein Synthesis (CFPS)

Another method of protein production and, hence, the incorporation of ncAAs into proteins is cell-free protein synthesis (CFPS). CFPS is a powerful and versatile platform as it involves protein production without the use of living cells, eliminating the cell membrane barrier and cell-viability constraints.^45,88−90^ This makes it suitable for the incorporation of more toxic ncAAs since the internal cell metabolism does not have to be preserved, as demonstrated by the global replacement of arginine with canavanine. Canavanine is a potent antimetabolite, and its presence in living cells causes cell death.^91^ Worst et al. have shown that, in a cell-free system, canavanine can be incorporated into recombinant proteins (GFP in this case) with high yields.^92^

**Figure 5:**
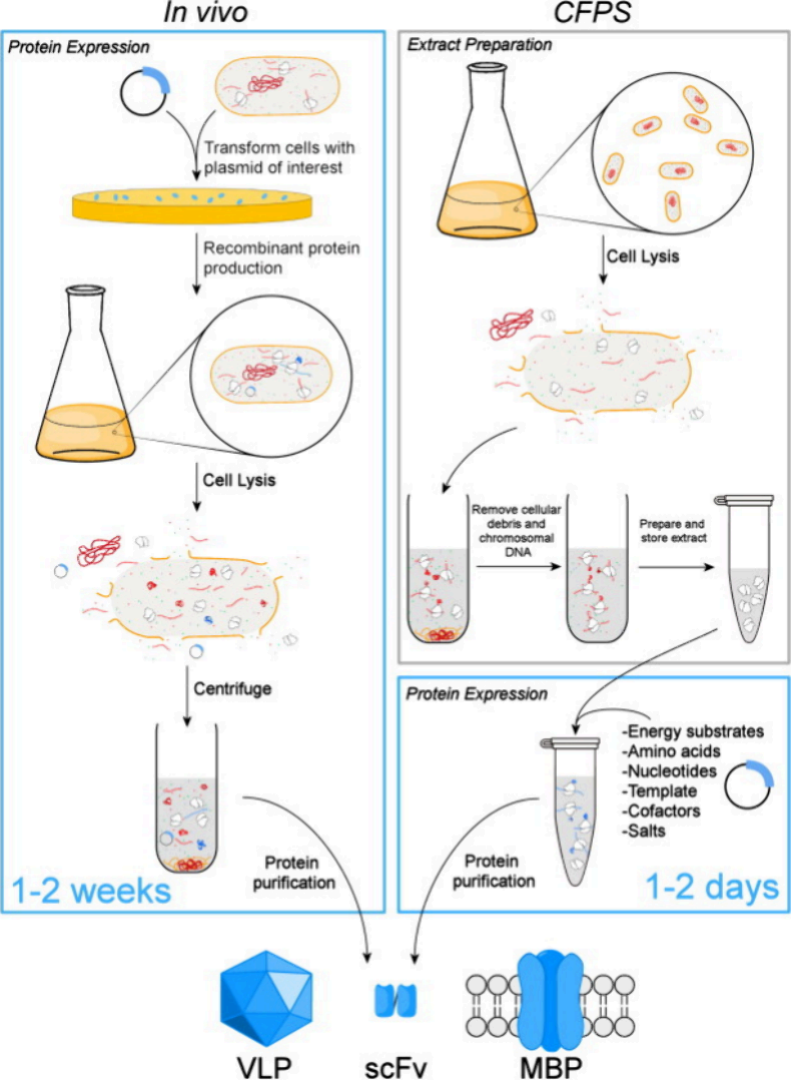
A comparison of cell-free and *in vivo* protein synthesis methods. Example proteins shown include a virus-like particle (VLP), a single-chain antibody variable fragment (scFv), and a membrane bound protein (MBP). Reprinted with permission from ref (93). Copyright 2011 Elsevier Ltd.

The CFPS process begins with the generation of the crude extracts of cultured cells^94^ (Figure 5). These extracts are depleted of endogenous DNA and mRNA and supplemented with energy components and free amino acids. The translational process is initiated by the addition of a suitable template (linear or circular DNA or mRNA).^88,95^ The conditions and components of CFPS systems are modified for each protein, depending on its complexity, folding, and required post-translational modifications. So, naturally, a wide range of CFPS systems have been produced, with *E. coli* being the most efficient platform.^15,96^ Several comprehensive reviews have been conducted on the different systems and applications of CFPS.^89,95,97−99^

For the RSI of ncAAs, the cell-free method cuts out the growth and starvation phases of expression, as the required ncAA only needs to be added to the expression mixture,^100,101^ making it a faster and more efficient process. Additionally, the open nature of the process means the protein production environment can be easily and directly modified especially when compared to *in vivo* protein synthesis, which must go through cloning and transformation steps. The main disadvantage of CFPS lies in its high cost, which stems from the need for energetic molecules like ATP and GTP.^102^ However, efforts are being made to reduce the costs through alternative ATP regeneration systems and the optimization of working reagents and concentrations.^103^

### 3.4 Engineering Aminoacyl-tRNA Synthetases

Though not a specific method of ncAA incorporation, the success of any method hinges on the ability of the biological machinery to accept the ncAA, specifically the aminoacyl-tRNA synthetases (aaRSs). Most cells contain a distinct aaRS for each of the 20 cAAs. These enzymes catalyze the activation and ligation of the amino acid to its cognate tRNA, thus forming a pool of aminoacylated tRNA (aa-tRNA) in the cell that is used for protein synthesis.^74^ Many aaRSs can be polyspecific, where they can charge multiple amino acids, and this is exploited to incorporate ncAAs without altering the active site of the enzyme. Hartman has summarized this work, showing the structures of all known ncAA substrates for each of the 20 *E. coli* aaRSs.^104^ To expand the diversity of ncAAs that can be incorporated, the engineering of the biosynthetic machinery must first occur. Several methods have been implemented including (i) introducing additional copies of the natural aaRS,^105−107^ (ii) enlarging the aaRS binding pocket, and (iii) shrinking the editing domain of the aaRS.^74,79,108^

Kiick et al. demonstrated that the *in vitro* activation rates of a series of Met analogues by the methionyl-tRNA synthetase (MetRS) correlated strongly with the ability of these amino acids to support protein synthesis *in vivo.*(74,109) So, the elevation of the aaRS activity by introducing additional copies of the aaRS results in the ability of the expression host to incorporate some ncAAs. Tang and Tirrell demonstrated this in the incorporation of hexafluoroleucine into an artificial leucine zipper protein.^105^ They established that the elevation of Leucyl-tRNA synthetase (LeuRS) activity is required for the incorporation of hexafluoroleucine and only leucine auxotrophic cells with the ability to overexpress LeuRS could produce proteins containing hexafluoroleucine.

**Figure 6:**
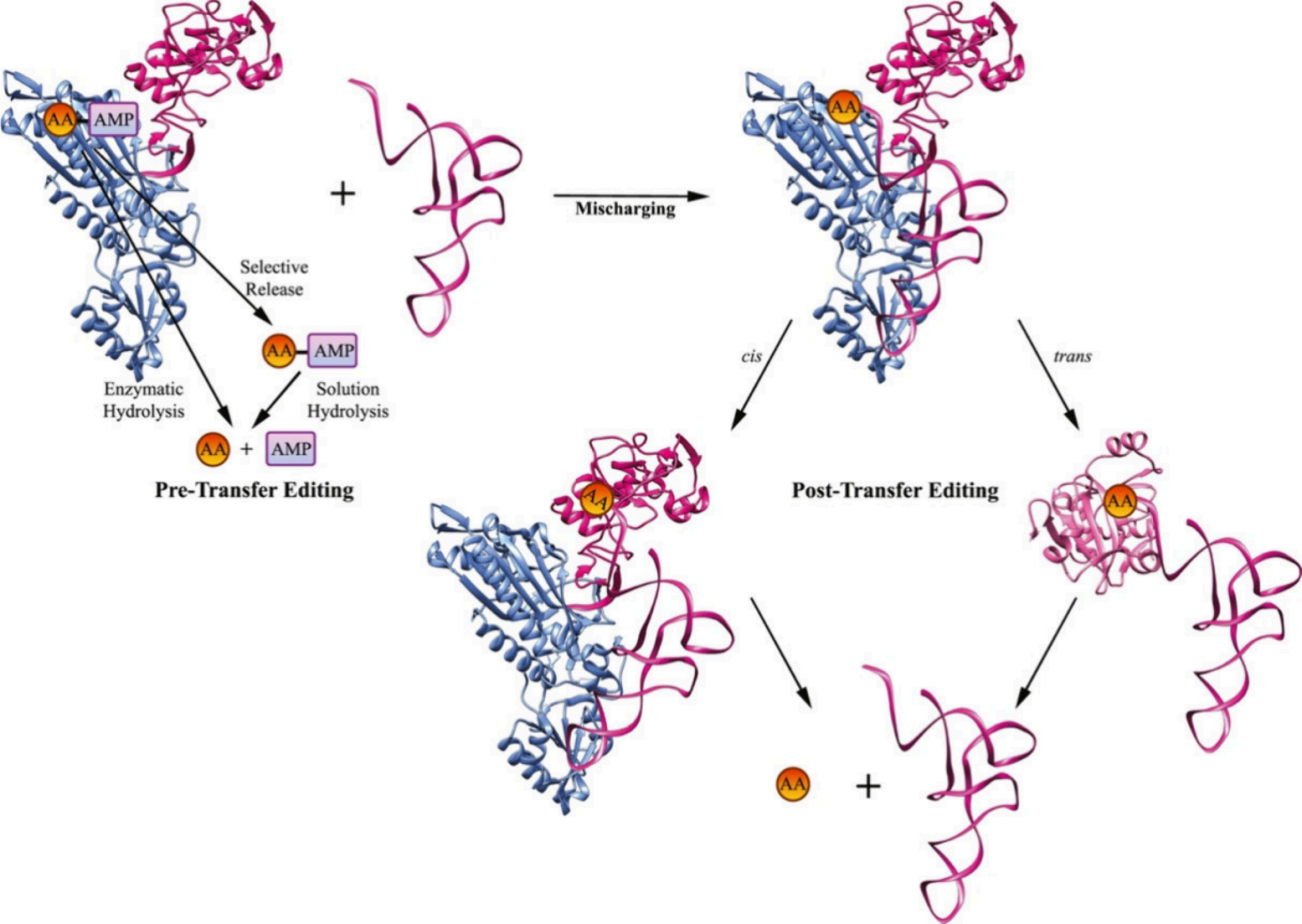
Aminoacyl-tRNA synthetase editing pathways. Once the incorrect amino acid has been activated forming an aminoacyl-adenylate (AA-AMP), pretransfer editing pathways will hydrolyze AA-AMP. The aminoacylation core of the aaRS, *E. faecalis* ProRS (represented as a monomer for simplicity) in this example, will either enzymatically hydrolyze the adenylate or release the adenylate from the active site where it will be hydrolyzed in the cytoplasm. Post-transfer editing occurs after the formation of mischarged aa-tRNA. Reprinted with permission from ref (112). Copyright 2020 Elsevier Ltd.

Additionally, engineering the aaRS itself can facilitate the incorporation of analogues that are poor substrates for the synthetase. The editing domain of aaRSs is responsible for clearing any improperly aminoacylated tRNAs. It ensures that only cognate amino acids are charged on tRNAs, hydrolyzing any aminoacylated tRNAs it identifies as not being conjugated to the correct amino acid, maintaining the fidelity of the translation process^110^ (Figure 6). Introducing certain mutations into the editing domain relaxes its specificity, allowing the incorporation of more ncAAs.^53^ Tang and Tirrell have shown that a T252Y mutation in the editing domain of *E. coli* LeuRS reduces its proofreading efficiency and allows the incorporation of a variety of Leu analogues that were previously not translationally active.^111^

Similarly, the specificity of aaRSs could be relaxed by changing the shape of its binding pockets. Kirshenbaum et al. showed that the introduction of a single alanine to glycine (A294G) mutation in the binding pocket of *E. coli* phenylalanine-tRNA synthetase (PheRS) allowed the incorporation of a variety of reactive Phe analogues.^113^ These methods have been employed to engineer a plethora of aaRSs.^114^

Additionally, several high-throughput screening methods have been developed for the identification of new aaRSs that enable RSI^115−117^ and SSI.^55,118,119^ Recently, Van Deventer and Stieglitz developed a method for the high-throughput screening of aaRSs libraries for the incorporation of ncAAs in yeast.^120^

Overall, it is very well established that simple changes in the cell aminoacylation machinery coupled with control of the amino acids available to the host leads to altered interpretations of the genetic code, facilitating ncAA incorporation.^74^ A particularly noteworthy development is the creation of flexizymes, which are small ribozymes capable of generating aa-tRNAs by the Suga group.^121,122^ It is a highly flexible tRNA aminoacylation system that tolerates a wide variety of tRNAs and is currently being used for aaRS-free cell-free translation systems.^123,124^

The incorporation of ncAAs into proteins and peptide materials encompasses a variety of techniques, from RSI to SSI and aaRS engineering. Collectively, these techniques contribute to the creation of a diverse range of proteins and peptides, driving innovation across a wide array of applications.^35^ Notably, RSI plays a pivotal role in labeling, cross-linking, fluorination, and enzyme engineering applications, as is elaborated in the following sections.

## 4 Applications of Residue-Specific Noncanonical Amino Acid Incorporation

### 4.1 Bio-orthogonal Noncanonical Amino acid Tagging (BONCAT), Fluorescent Noncanonical Amino acid Tagging (FUNCAT), and Threonine-Derived Noncanonical Amino Acid Tagging (THRONCAT)

**Figure 7:**
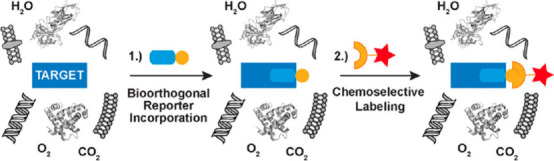
Two-step approach for chemoselective biomolecule labeling: In the first step, a functional group (orange circle), covalently attached to a substrate (light blue rectangle), is introduced into a biomolecule of interest by genetic or chemical methods. In a second step, the functional group reacts chemoselectively, in the presence of all the functional groups found in a living cell or animal, with an added chemical probe (orange arc with red star). Gray shapes denote other biomolecules that must not react with the chemical reporter or the external chemical probe. Reprinted from ref (53). Copyright 2014 American Chemical Society.

Biomolecular labeling involves incorporating a unique chemical functionality into a molecule of interest and then reacting this functionality with an external chemical probe in a specific manner^53^ (Figure 7). Labeled proteins can be selectively conjugated to fluorophores, affinity reagents, peptides, nanoparticles, or surfaces for a wide variety of downstream applications in proteomics and biotechnology.^19^ These incorporated functionalities and probes must not significantly alter the other functional groups in the protein; they must be bio-orthogonal for any obtained data to be useful.^125^

Bio-orthogonal chemistry, as introduced by Bertozzi and colleagues in their work on the labeling of mucin-type O-linked glycoproteins,^126^ refers to reactions that can take place in biological environments without affecting biomolecules or interfering with biochemical processes.^127^ There are a wide range of bio-orthogonal reaction classes as outlined in several reviews.^127−130^ ncAAs offer additional bio-orthogonal handles for protein labeling, enabling scientists to explore a wider range of labeling strategies and applications.^131,132^ Lang and Chin have provided a comprehensive review focusing on the range of bio-orthogonal functionalities available with ncAAs.^53^

Bio-orthogonal noncanonical amino acid tagging (BONCAT) was formally introduced by Dieterich and colleagues^133^ as a method for visualizing newly synthesized proteins in mammalian cells. It has since been used in bacteria,^46,134,135^ in archaea,^136^ in microbial cultures,^137−139^ in plant systems,^140^ and even for *in vivo* labeling in living organisms like fruit flies^141^ and mice.^142,143^ BONCAT involves the incorporation of an azide or alkyne functionality into proteins that then can undergo click chemistry.^53^ Click chemistries refer to a set of chemical reactions that are readily catalyzed in aqueous solutions at atmospheric pressure and biologically compatible temperatures, with few toxic intermediates, and relatively fast reaction kinetics.^19,53,144^ The azide–alkyne cycloaddition reaction is most widely used for BONCAT even though azide–phosphine reactions have also been demonstrated.^109^

**Figure 8:**
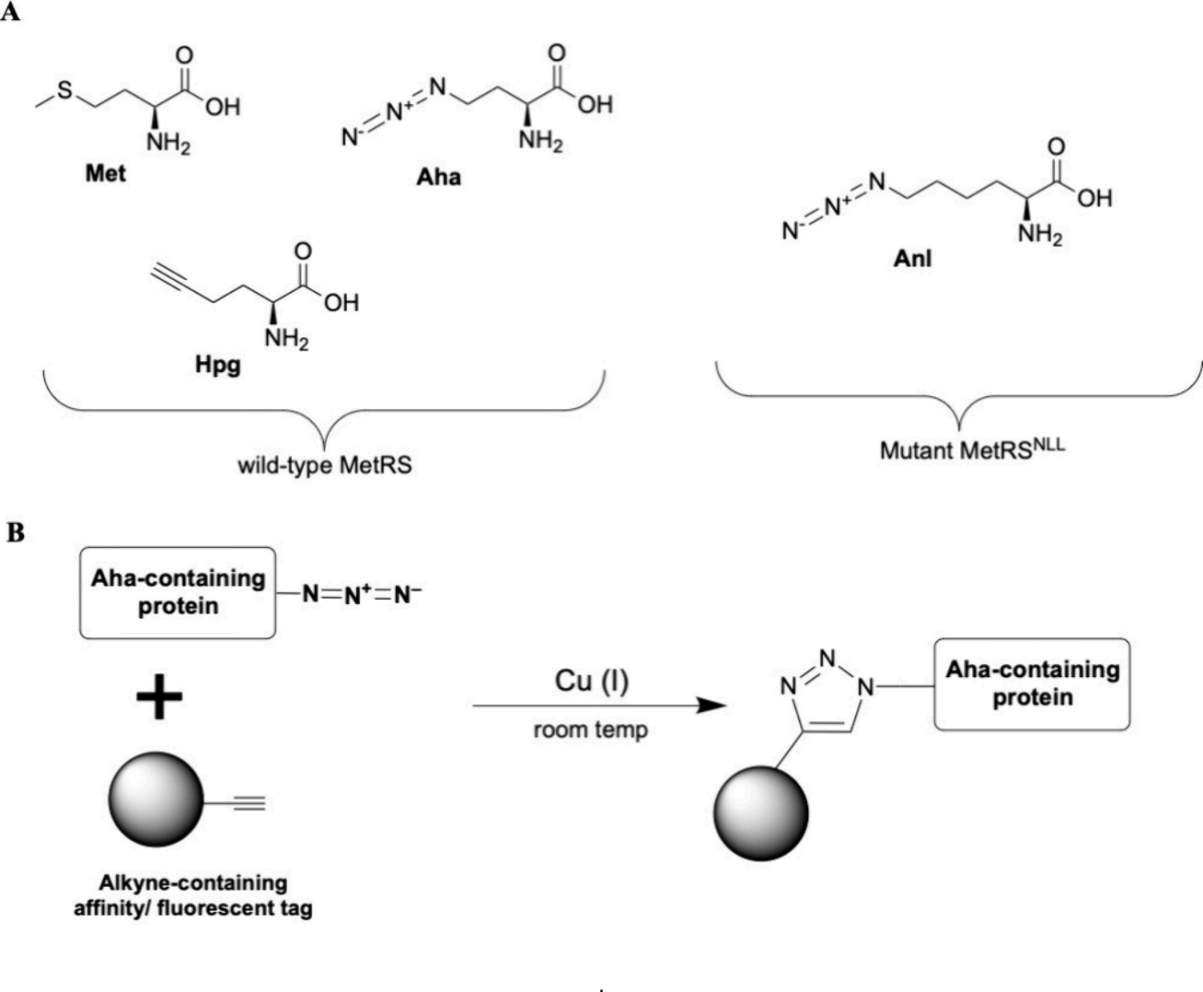
(A) The structures of methionine (Met) and methionine analogues: homopropargylglycine (Hpg), azidohomoalanine (Aha), and azidonorleucine (Anl). They can all be charged to tRNA^Met^ by the wild-type MetRS, apart from Anl, which can be utilized only by a mutant synthetase like *E. coli* MetRS^NLL^. (B) Reaction scheme of a click cycloaddition between a protein that has incorporated Aha and an alkyne conjugated to an affinity/fluorescent tag.

**Figure 9:**
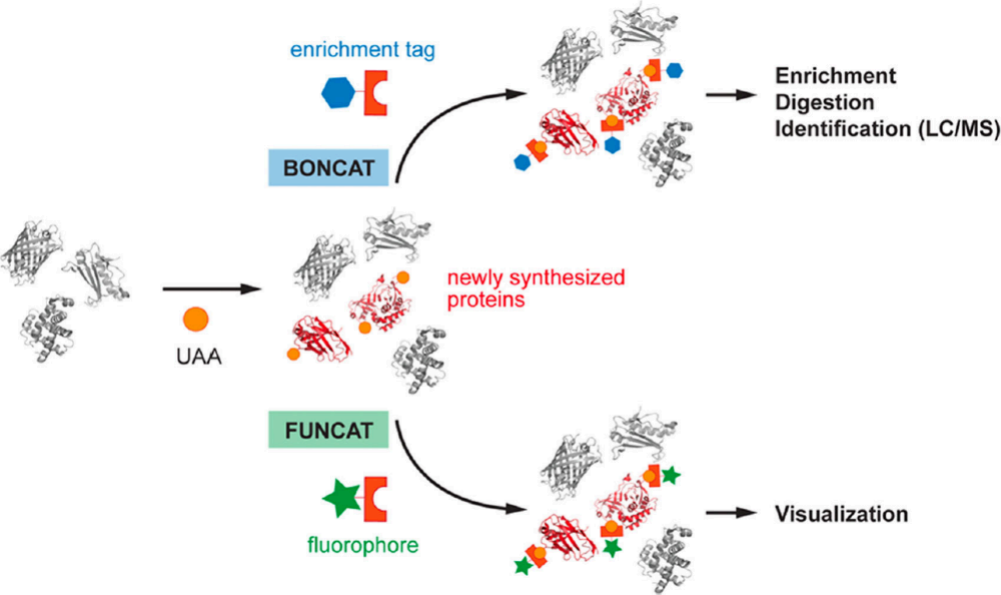
BONCAT and FUNCAT. An ncAA (orange sphere) is incorporated residue-specifically into newly synthesized proteins. ncAA-tagged proteins can be ligated to affinity probes for enrichment and identification (BONCAT) or to dyes for visualization by in-gel fluorescence scanning or fluorescence microscopy (FUNCAT). Reprinted from ref (53). Copyright 2014 American Chemical Society.

The process typically involves the replacement of methionine residues in the protein of interest with azidohomoalanine (Aha), azidonorleucine (Anl), or homopropargylglycine (Hpg) (Figure 8, Table 1). The use of *p*-azido-l-phenylalanine (Azf) to replace phenylalanine has also been demonstrated.^145^ These ncAAs contain either an azide group (Aha, AnI, and Azf) or an alkyne group (Hpg), which can be covalently coupled to an alkyne or azide-functionalized enrichment tag and analyzed via mass spectroscopy. The newly synthesized protein will contain the ncAA and enrichment tag, enabling it to be analyzed (Figure 9). While the substitutions can have a negative impact on cellular protein synthesis, indeed Hpg has been shown to negatively impact bacterial metabolism^146,147^ and Aha can inhibit plant protein cell growth rates,^148^ methods have been developed to mitigate these effects.^149,150^

**Table 1:** Recent Applications of BONCAT, FUNCAT, and THRONCAT Labeling Techniques

ncAA incorporated	Function	Labeling	Ref
Aha	Perform proteomic analysis of *L. mexicana*. Quantitative profiling of the changes in the synthesis of hundreds of parasite proteins as functions of dose and duration of the inhibitor treatment.	Liquid chromatography–mass spectrometry; fluorescence	(48, 168)
Aha	Quantify the amount of newly synthesized proteins in the study of the antiviral activity of aminoglycoside Geneticin against SARS-CoV-2 variants.	Fluorescence	(50)
Aha	Determine the effect of Aha treatment on protein abundance in HeLa cell cultures.	Liquid chromatography–mass spectrometry	(150)
Aha, Hpg	Identify newly synthesized proteins in mice.	Fluorescence	(142)
Aha	Visualize and quantify bacterial translational activity in expectorated sputum from cystic fibrosis lungs.	Fluorescence	(161)
Aha	Analyze the response of lung cancer cells to cisplatin.	Mass spectroscopy	(172)
Aha	Measure rapid and sustained protein synthesis dynamics in the retina and subsequent transport of newly synthesized proteins to the optic nerve before and after injury.	Fluorescence; mass spectroscopy	(173)
Aha	Determine the degradation rate (half-life) of cellular protein	Fluorescence; Western blot analysis	(183)
Aha	Determine the proteome-wide translation rates in engineered 16S rRNAs (rRNAs) from *E. coli*, *P. aeruginosa*, and *Vibrio cholerae*	Fluorescence	(184)
Anl	Analyze the metabolic activities and response to antibiotics of a specific subpopulation of *P. aeruginosa*	Fluorescence; mass spectroscopy; Western blot analysis	(46)
Anl	Demonstrate cell-selective analysis of protein synthesis in hamster (CHO), monkey (COS7), and human (HeLa) cell lines.	Fluorescence; mass spectroscopy	(185)
Anl	Study the protein synthesis dependent processes in *D. melanogaster*	Fluorescence; Western blot analysis	(141)
Anl	Analyze the type III secretion system of the human pathogen *Y. enterocolitica* and identify distinct secretion profiles for intracellular and extracellular bacteria.	Fluorescence; Western blot analysis	(164)
Anl	Proteomic analysis of *B. thailandensis* to determine its profile as it infects host cells and the proteins that are differentially regulated in infection conditions.	Fluorescence; mass spectroscopy; Western blot analysis	(165)
Anl	Identify the proteins that were altered during spatial memory formation in hippocampal neurons of a mouse strain.	Fluorescence; mass spectroscopy; Western blot analysis	(143)
Azf	Label proteins with spatiotemporal selectivity in live worms, *Caenorhabditis elegans*, for the discovery of proteins uniquely expressed in a subset of cells.	Liquid chromatography–mass spectrometry; fluorescence	(145)
βES (THRONCAT)	Visualize and enrich nascent proteins in bacteria (*E.coli*), mammalian cells (HeLa), and *D. melanogaster*.	Liquid chromatography–mass spectrometry; fluorescence	(175)
Hpg	Image, capture, and interrogate shigatoxigenic/verotoxigenic *E. coli* (VTEC) and *Listeria*.	Fluorescence	(134, 135)
Hpg	Assess the diversity and activity of microbial communities from glacial surfaces in Iceland and Greenland.	Fluorescence	(138)
Hpg	Identify anabolically active members of a microbial community incubated in the presence of various growth substrates and under changing physicochemical conditions.	Fluorescence-activated cell sorting and 16S rRNA gene amplicon sequencing	(139)
Hpg	Detect and quantify active antimicrobial-resistant bacteria in water samples.	Fluorescence	(155, 186)
Hpg	Investigate the changes in the translational activity of nitrite-oxidizing bacteria populations throughout batch exposure to free ammonia.	Fluorescence-activated cell sorting	(156)
Hpg	Identify translationally active cells in sludge.	Fluorescence-activated cell sorting and 16S rRNA gene amplicon sequencing	(157, 158)
Hpg	Quantify active populations in aquatic environments.	Fluorescence	(159)
Hpg	Probe and identify the translationally active members of the gut microbiota.	Fluorescence-activated cell sorting and 16S rRNA gene amplicon sequencing	(160)
Hpg	Measure the translationally active cells in soil.	Fluorescence	(187)

Fluorescent noncanonical amino acid tagging (FUNCAT) involves a similar process where the ncAAs are covalently coupled to azide or alkyne bearing fluorescent dyes instead of enrichment tags allowing the labeled proteins to be visualized by fluorescence microscopy (Figure 9). This simplifies analysis as the newly synthesized proteins can be easily visualized, quantified, and imaged.^151,152^ Varricchio et al.^50^ have used this method in their study of the antiviral activity of Geneticin against SARS-CoV-2 variants. Geneticin is an aminoglycoside that has shown antiviral activity against bovine viral diarrhea and hepatitis C virus (HCV).^153,154^ FUNCAT is used to quantify the amount of newly synthesized proteins in the Geneticin treated cells to determine that antiviral activity is occurring in the absence of cell toxicity.^50^ Similarly, the method has been used to detect and quantify translationally active cells in water samples,^155^ glacial surfaces,^138^ activated sludge,^156−158^ aquatic environments,^159^ and microbiota^160,161^ (Table 1).

BONCAT and FUNCAT have become staples of the proteomics toolkit for the detection and identification of proteins synthesized within defined time intervals.^19,49,53,140,150,162,163^ By using an ncAA that is not incorporated into proteins by the endogenous translational machinery like Anl, which is only incorporated in the presence of a mutant methionyl-tRNA synthetase, NLL-MetRS,^116,164^ specific cell populations can be targeted and studied.^43,145,164,165^ For example, the Tirrell group has explored the use of Anl incorporation in the study of the type III protein secretion profiles of of the human pathogen *Yersinia enterocolitica.*(164) Babin and colleagues have also used this method to analyze a distinct subpopulation of a *Pseudomonas aeruginosa* biofilm that showed increased tolerance to antibiotics. They selectively label, enrich, and identify proteins expressed by cells within distinct regions of a biofilm, finding that the labeled subpopulation is characterized by a lower abundance of ribosomal proteins and was enriched in proteins of unknown function.^46^

BONCAT has been very useful for the study of newly synthesized proteins *in vivo*. Calve et al. demonstrated that Aha and Hpg can be globally incorporated into mice through facile intraperitoneal injections.^142^ The Götz lab has since used this to identify *de novo* proteomic changes which occur in the hippocampal neurons of mice during spatial long-term memory consolidation.^143^ By engineering a mouse strain that restricts Anl labeling to just the hippocampal neurons, they have identified 156 proteins that are altered in synthesis during spatial memory formation. They have also used Aha incorporation to determine the effect of Tau, a scaffolding protein, on protein synthesis in the K369I tau transgenic K3 mouse model of frontotemporal dementia.^166^ Similarly, Erdmann et al. have demonstrated that Anl can be incorporated into fruit flies (*Drosophila melanogaster*) through their diet after engineering the drosophila MetRS, allowing for the study of protein-synthesis-dependent processes in the organism.^141^

**Figure 10:**
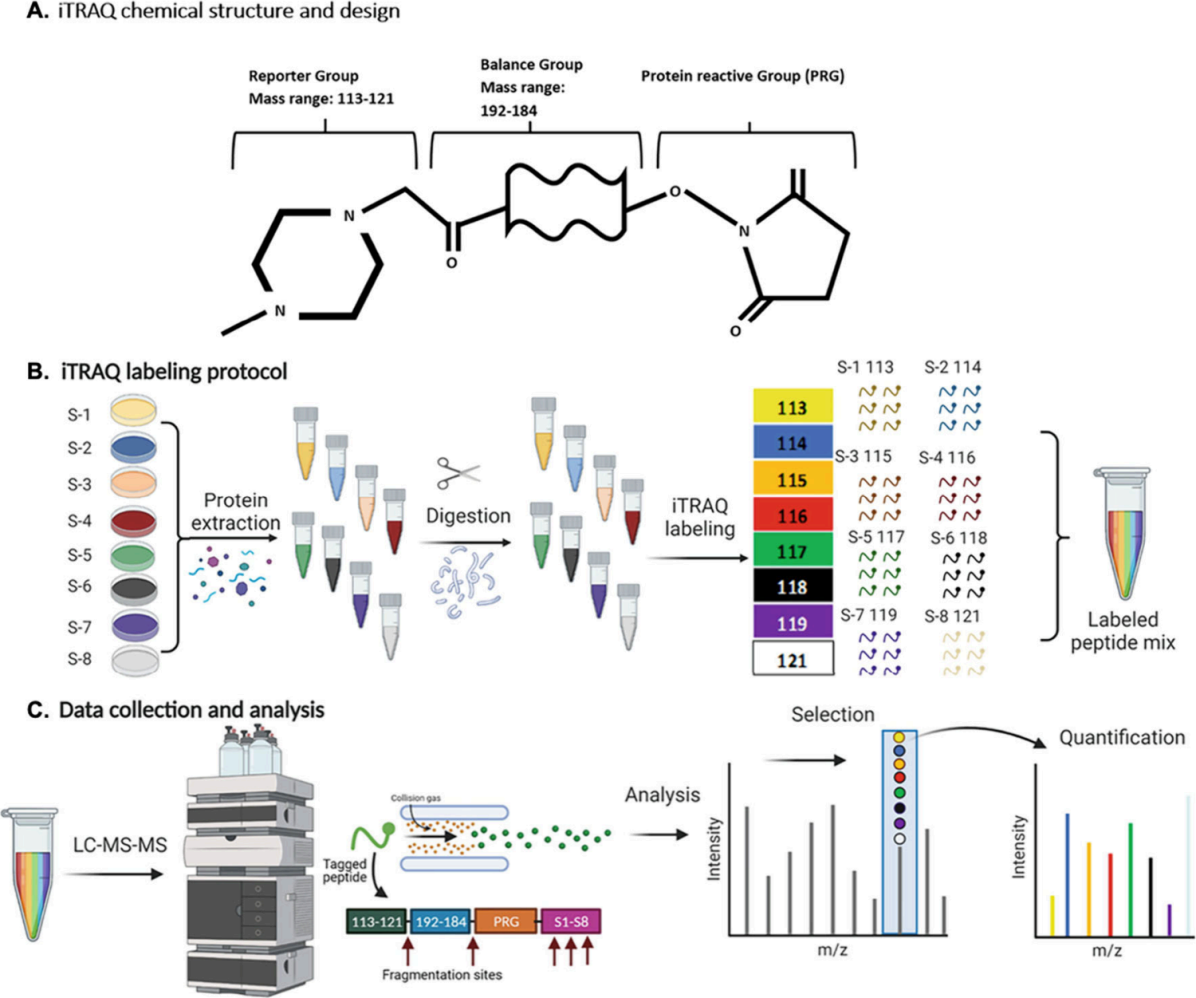
(A) iTRAQ chemical structure and design. In an 8-plex iTRAQ experiment, the total mass of the reporter group and the balance group is identical across all eight iTRAQ reagents. (B) After proteins are extracted and digested into peptides, they are labeled, each sample with a different tag. (C) During quantification, the mass spectrometer isolates the ionized peptide and records the mass of the intact labeled peptide (MS1), which is the same across all labeled samples for the same peptide. Then the peptide is fragmented, and the fragments’ masses are measured (MS2). This allows the relative quantification of the peptide across the eight samples. Reprinted with permission from ref (170). Copyright 2022 under exclusive license to Springer Science Business Media, LLC, part of Springer Nature.

BONCAT has also been used for the identification and analysis of protozoan parasite proteome dynamics.^135,165,167^ Kalesh et al. have used a combination of BONCAT and isobaric tags for relative and absolute quantitation (iTRAQ) based quantitative proteomic mass spectrometry (Figure 10) for various studies on *Leishmania mexicana* (a causative agent of leishmaniasis, a parasitic disease affecting approximately 12 million people worldwide).^48,168^ Here, BONCAT is first employed to determine the starvation adapation mechanism of the parasite^168^ and then to provide the first protein-level evidence that heat shock protein 90 (Hsp90) inhibition affects global protein synthesis in *L. mexicana.*(48) By monitoring the effect of tanespimycin (an Hsp90 inhibitor) on the parasites, the specific dose- and time-dependent effects of tanespimycin treatment on the synthesis of several key parasite proteins are determined, information considered vital for the development of future antileishmanial drugs.^169^

The studies highlighted above demonstrate BONCAT’s potential in therapeutic research for understanding parasite behaviors and responses to potential treatments.^171^ Tangentially, BONCAT has also been used to monitor the responses of mammalian cells to injury and treatments.^172−174^ Monteiro and colleagues have used it to analyze the resistance of lung cancer cells to the drug cisplatin and determine the potential targets to monitor in epigenetic resistance in lung cancer.^172^ Shah et al. have also used it to measure the protein synthesis and transport dynamics in the retina before and after optic nerve injury. They have identified the genes that significantly modulate neurite growth *in vitro*, demonstrating the potential for BONCAT to identify candidate protein regulators of the degenerative and regenerative responses.^173^

**Figure 11:**
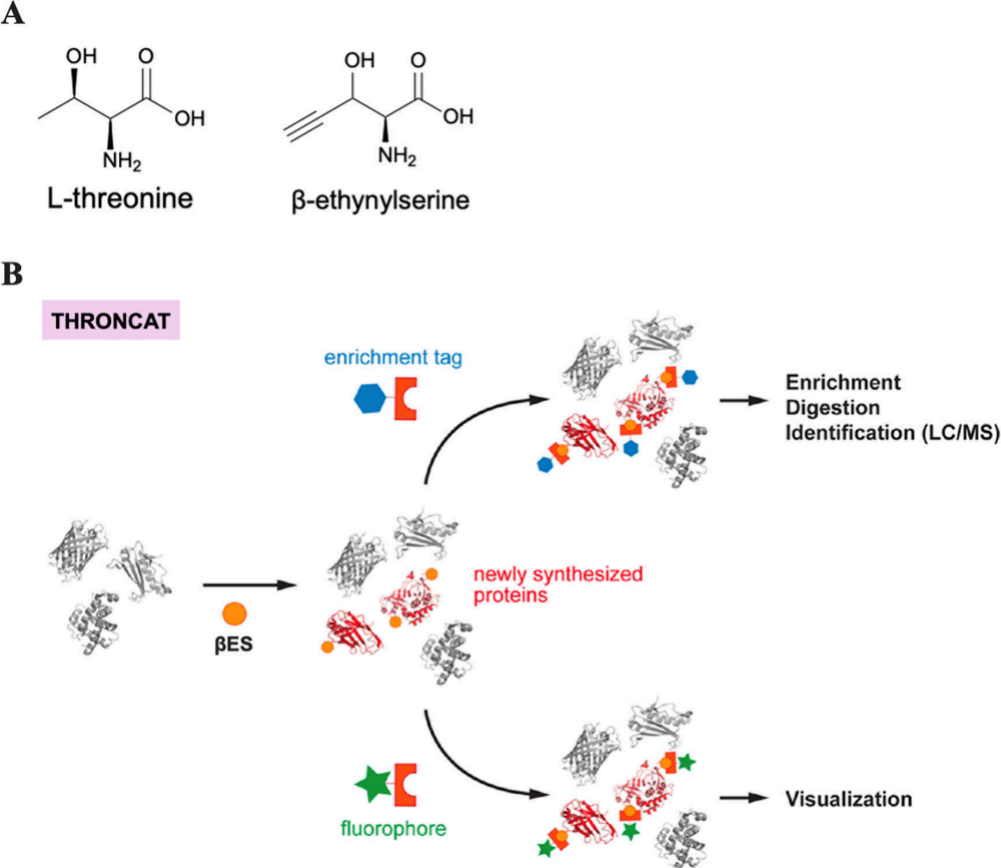
(A) The chemical structures of threonine and β-ethynylserine (βES). (B) THRONCAT. βES (orange sphere) is incorporated into newly synthesized proteins in complete growth media. βES-tagged proteins can be ligated to affinity probes for enrichment and identification or to dyes for visualization by in-gel fluorescence scanning or fluorescence microscopy. Adapted from ref (53). Copyright 2014 American Chemical Society.

Recently, the Bonger lab introduced threonine-derived noncanonical amino acid tagging (THRONCAT), which involves the replacement of threonine with its analogue β-ethynylserine (βES) in complete growth media without auxotrophic cell lines.^175^ βES contains an alkyne group which allows newly synthesized proteins to be covalently coupled to azide-functionalized enrichment or fluorescent tags and analyzed, similar to BONCAT and FUNCAT (Figure 11). They observed that the labeling occurs in complete growth media throughout the proteome with a 12.5-fold higher incorporation rate compared to Hpg. They use the method for the visualization and enrichment of nascent proteins in HeLa cells and for the *in vivo* quantification of protein synthesis rates in *D. melanogaster*. This is a very notable advancement as THRONCAT does not require auxotrophic cell lines or threonine-free conditions, increasing the scope of cell lines amenable to metabolic protein labeling and making the process less laborious. Also, in cases where exhaustive identification of proteins is required, THRONCAT can be used in tandem with BONCAT to increase proteomic coverage.

Finally, SSI has also been widely popular for labeling applications due to its ability to attach an organic probe to a specific protein position.^176,177^ For a similar effect, RSI would require POI-encoding DNA to undergo mutagenesis to remove extraneous ncAA sites. Without their removal, off-target inhibition of protein function or aggregation can be causes of concern. BONCAT in SSI also possesses inherent advantages over other covalent labeling methods such as N- or C-terminal conjugation techniques^178−180^ which can disrupt overall protein function.^181,182^ Both RSI and SSI have their unique strengths, and the choice between them should be guided by the requirements of the desired application.

RSI for BONCAT and FUNCAT applications allows for facile labeling, which makes it a powerful and versatile technique. This versatility is further demonstrated in some of its recent applications shown in Table 1.

### 4.2 Cross-Linking

The incorporation of ncAAs equipped with cross-linking capabilities has found application in the study of protein interactions and development of new materials.^188^ Cross-linking is a process where two or more molecules or domains are chemically or physically joined, and the methods commonly used are photo-cross-linking, where bonds are created by UV irradiation of photoactivatable groups,^189,190^ and chemical cross-linking, where covalent bonds are formed between specific reactive groups.^191,192^ These methods offer various avenues for studying protein structure and interactions as well as for the creation of new materials.

The molecular cross-linking of proteins can be used to create matrixes with desired mechanical and functional properties without being toxic to the host system.^191^ Finkler et al. have fully replaced Lys with hydroxylysine in green fluorescent protein, giving the protein cross-linking capabilities.^45^ Hydroxylysine is an important component of collagen fibers produced by post-translational modification of Lys residues. In collagen, the enzyme lysyl oxidase (LOX) catalyzes the formation of aldehydes from Lys or hydroxylysine residues. These aldehydes then condense with one another to form pyridinoline cross-links.^193,194^ It has been demonstrated that supplementing tissue-engineered neocartilage with hydroxylysine and copper sulfate significantly increases its pyridinoline cross-links, resulting in a 3.3-fold increase in the tensile strength of the material.^195^ Direct incorporation of hydroxylysine is a step to producing synthetic collagen without the need for animal sources.^196^

Zhang et al. have produced a polypeptide scaffold for protein immobilization using *p*-azidophenylalanine (pAzF), an ncAA that generates covalent linkages to substrates upon UV irradiation.^197^ By using a mutant *E. coli* phenylalanyl-tRNA synthetase (A294G), they partially replace the phenylalanine residues in their polypeptide with pAzF, producing a material that can effectively immobilize recombinant proteins.^198^ This innovation opens up possibilities for immobilizing antibodies and enzymes for biosensors and other diagnostic applications.^199^

Furthermore, several groups have used pAzF with their ELP domains to create globular protein vesicles with tunable structural integrity and size. The resulting vesicles show high potential for small molecule/protein combinations or targeted therapies.^200,201^ Carrico et al. have also incorporated pAzF into their artificial extracellular matrix proteins, allowing the production of films that can be directly patterned using photolithographic techniques.^202^

It is important to note that bio-orthogonal conjugation may have an impact on protein supramolecular structure. To avoid negative effects of large peptide conjugations and retain protein secondary structure, Montclare and colleagues have utilized bissulfosuccinimidyl suberate (BS^3^) ester cross-linking (X) to maintain protein oligomerization of an Aha-incorporated coiled coil, Q_Aha_, for supramolecular assembly into fibers.^203^ Following small molecule binding and cross-linking, negligible differences in fiber morphology were noted, and the construct was allowed to undergo copper-catalyzed click chemistry with relatively large iron templating peptide CMmS6. Upon biomineralization of ultrasmall superparamagnetic iron oxide (USPIO) nanoparticles, the protein conjugate, Q_Aha_-X-CMmS6-USPIO, was capable of sensitive *T*_2_*-weighted MRI imaging due to dense USPIO templation.

**Figure 12:**
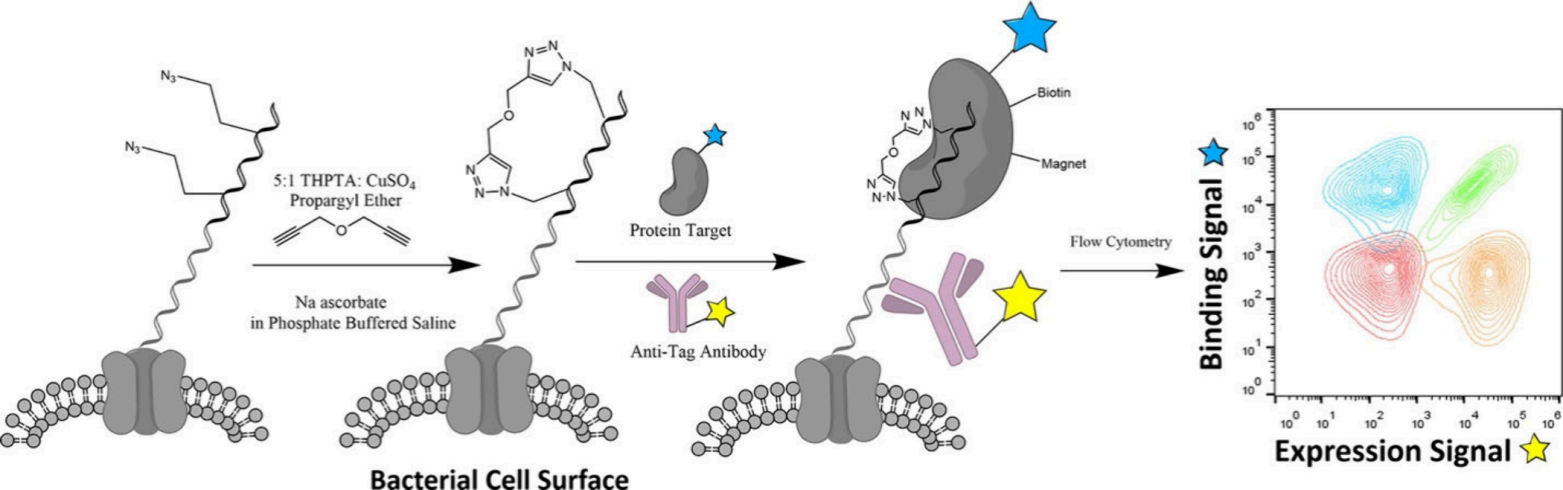
Stabilized peptide engineering by *E. coli* display (SPEED). DNA encoding peptide is transformed into *E. coli* and expressed on the cell surface by incubating bacteria in an azide-containing methionine analogue. After click chemistry is performed directly on the cell surface, bacteria are incubated with fluorescent epitope tag antibody and protein target. Finally, bacterial cells are analyzed via flow cytometry. Reprinted from ref (204). Copyright 2023 American Chemical Society.

Cross-linking has also been used to stabilize the secondary structures of proteins and peptides. Peptide stapling is a technique that uses the chemical cross-linking of two residues on the same or different peptides to constrain the conformation of the peptides. This constraint has been shown to improve the peptide’s target affinity and enhance its proteolytic stability.^205−208^ The Thurber lab has developed a peptide screening method called Stabilized Peptide Evolution by *E. coli* Display (SPEED), which involves the display of stabilized peptides on the surface of methionine auxotrophic *E. coli.*(209) Aha is incorporated into the displayed peptide through RSI, and the resulting protein is reacted with a stabilizing bis-alkyne cross-linker via copper-catalyzed click chemistry. The stabilized peptide is then incubated with the target protein, which is biotinylated or fluorescently labeled, allowing the cells to be analyzed via flow cytometry (Figure 12). This method has been used to measure the impact of staple chemistry and staple location on affinity peptides for the murine double minute-2 (mdm2) and B cell lymphoma 2 (Bcl-2) family of proteins.^204^ SPEED is used to design novel stapled peptides in both systems, demonstrating that the discovery of stapled peptides via bacterial surface display is a powerful method that can optimize sequence, staple location, and staple chemistry with respect to binding affinity and specificity.

**Figure 13:**
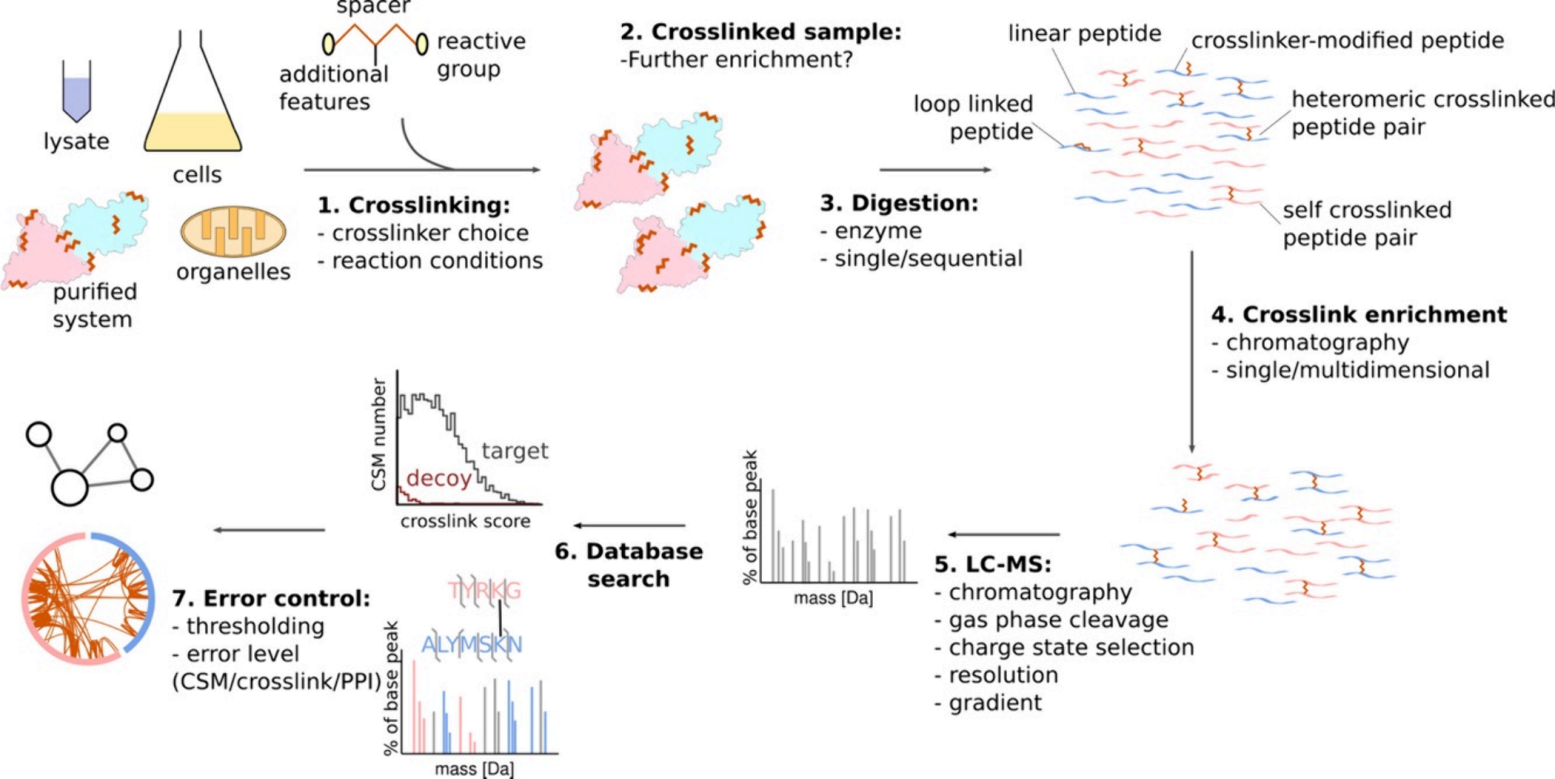
Cross-linking mass spectrometry workflow and experimental design. Step 1. Samples of varying degrees of complexity are reacted with soluble cross-linkers. These introduce covalent bonds between residues that are close in space. After cross-linking, the sample may be further (step 2) purified biochemically or (step 3) directly subjected to digestion. Sequential or parallel digestion strategies may be employed for more complex samples. The resulting peptide mixture contains cross-linked peptide pairs, loop-linked peptides (where the cross-linker reacted within the peptide), cross-linker-modified peptides, and linear peptides, in order of abundance. Step 4. Cross-linked peptides may then be enriched by a single or multiple rounds of chromatography. Step 5. The resulting samples are acquired by LC–MS using specific methods designed to detect and fragment cross-linked peptide pairs and (step 6) computationally searched and matched against a sequence database, yielding cross-link spectrum matches (CSMs). Step 7. Finally, the appropriate error control is applied to derive a false discovery rate for the data set at the level of CSMs, residue pairs, or protein–protein interactions (PPIs). Reprinted with permission from ref (210). Copyright 2021 Elsevier Ltd.

Finally, cross-linking mass spectrometry (XL-MS) is an emerging technique to obtain structural information on biomacromolecules and their complexes *in vivo* and *in vitro*(210−216) (Figure 13). The general principle is that nearby protein residues can be cross-linked and, after digestion, those cross-linked residues can be identified, thus revealing their proximity in the original sample. The maximum length of the cross-linker acts as a distance constraint. By using these distance constraints as the basis for computational modeling, protein 3D structures as well as information on their folding and the topology of their complexes can be derived. Certain photoreactive amino acids can be induced to cross-link and then be analyzed using XL-MS to provide unique short-distance information on the structural core regions of proteins. Kohl et al. demonstrate high-yield incorporation of photoleucine (replacing leucine) into proteins recombinantly expressed in *E. coli* and analyzed using this method.^217^ The resulting structural data agreed with published structures of the sample protein, highlighting the effectiveness of this approach.

Overall, the ability to form cross-links is a versatile functionality that can be used to create new materials or study the structure of existing ones. SSI has also been used to give proteins cross-linking abilities, primarily to study specific protein–protein interactions with high precision and sensitivity.^218,219^ RSI is more conducive for the development of new materials though, as demonstrated by the XL-MS technique, can be used to provide important structural data.

### 4.3 Fluorination

Fluorine-bearing ncAA residues play a crucial role in protein research, as they allow for fluorine-based detection using ^19^F nuclear magnetic resonance (NMR) spectroscopy and impact protein structure and thermostability.

NMR spectroscopy is an established method for analyzing protein structure, interaction, and dynamics in different states.^220−222^ Here, a magnetic field is applied to a protein construct and the relative relaxation energy allows for a characteristic resonance frequency of its atomic nuclei according to its chemical or environmental surroundings.^223^ Fluorine is a favored NMR probe due to its small size, strong inductive effect, and near total absence in biological systems.^224,225^ In fact, while ^19^F exists in 100% natural abundance, it is nearly absent in organisms and there have only been five identifications of natural products containing organic bound fluorine.^226^ This means specific protein structures and interactions can be monitored with no interference from background signals, making it an effective probe as the derived signal is directly proportional to the concentration of ^19^F that has been inserted into the compound.^227−229^

Incorporating fluorinated residues into a protein allows its interactions to be studied via ^19^F NMR.^230−234^ Recently, Vogel and colleagues have employed RSI of ncAAs to determine the role of tyrosine residues in the antimicrobial peptide, tritrpticin. *o*-, *m*-, and *p*-fluorophenylalanines (fFs), 2-fluorotyrosine (2fY), and 3-fluorotyrosine (3fY) have been incorporated into tritrpticin via RSI where the cells are grown in the presence of glyphosate, an aromatic amino acid biosynthesis inhibitor.^235^ SDS micelles have been used to study the peptide–membrane interactions as they resemble the overall negatively charged membrane surface of bacteria. Solvent-perturbation ^19^F NMR measurements reveal that *p*-fF residues are embedded deeply in the hydrophobic region of the micelles, 3fY residues are located near the surface of the micelles with high solvent exposure, and 2fY side chains are less solvent exposed. Combined with the antimicrobial activity of the respective tritrpticin variants, the ^19^F NMR results indicate that higher solvent exposure of tyrosine residues corresponds to a decrease in antimicrobial potency.

**Figure 14:**
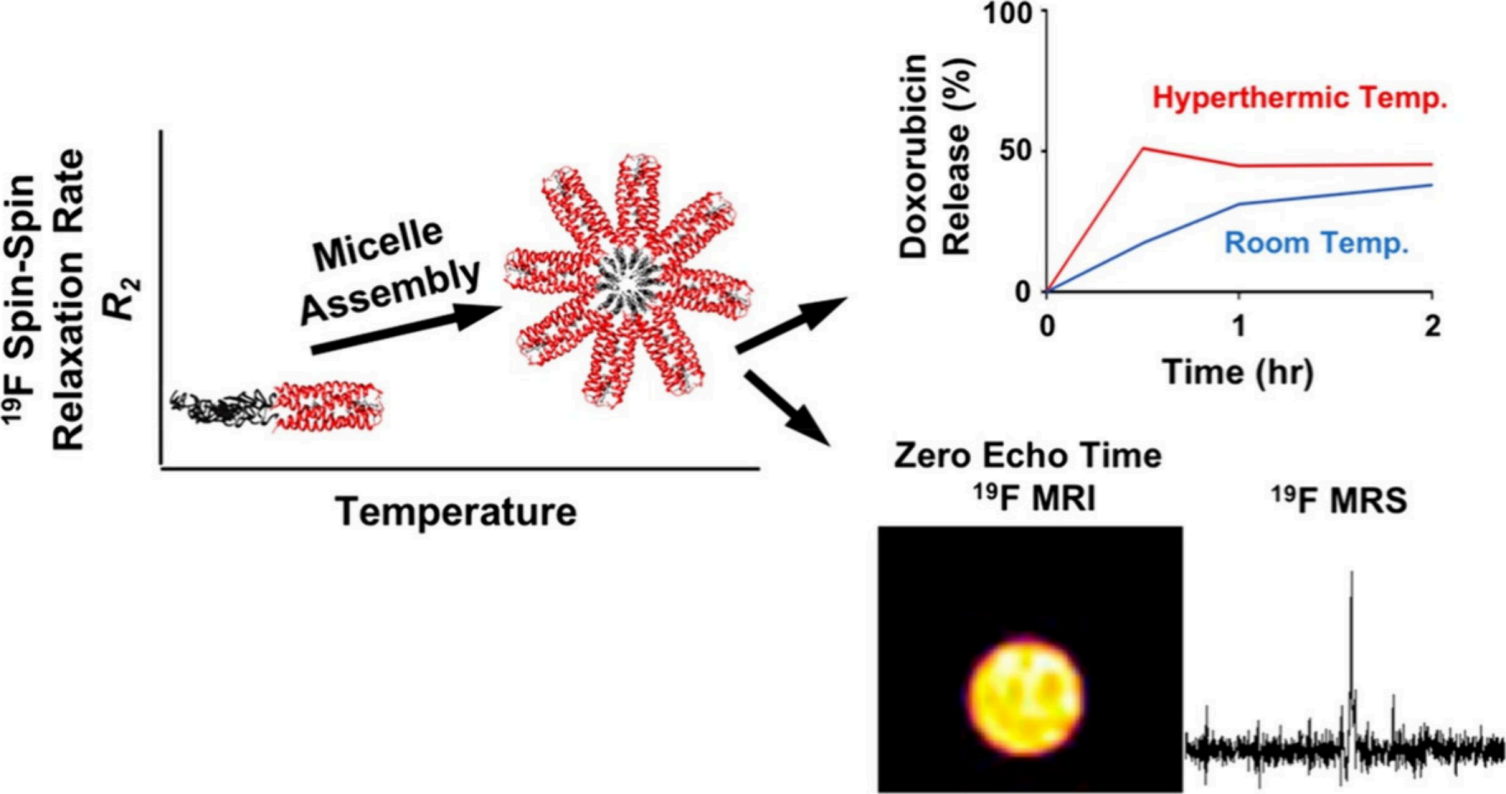
Schematic representation of F-TRAP assembly, thermoresponsive drug release, and detection by ^19^F MRS and by zero-echo time (ZTE) ^19^F MRI when sufficient fluorine is present. Reprinted from ref (228). Copyright 2019 American Chemical Society.

Similarly, Hill et al. have produced a protein block copolymer that allows for both ^19^F NMR and ^19^F magnetic resonance spectroscopy (MRS).^228^ Thermoresponsive assembled protein (TRAP) is a block copolymer with a coiled-coil domain that binds an array of small molecules^236−238^ including doxorubicin (Dox), an anthracycline-class chemotherapeutic agent that has broad therapeutic efficacy against an array of tumor types.^239^ By replacing the Leu residues with 5,5,5-trifluoroleucine (TFL), they have created fluorinated thermoresponsive assembled protein (F-TRAP), which assembles into monodispersed nanoscale micelles that can be detected by ^19^F NMR and *in vivo* by ^19^F MRS, presenting an avenue for the development of traceable theranostic agents (Figure 14).

Aside from its use in imaging techniques, fluorination stands out as one of the most powerful tools for the enhancement and stabilization of proteins. The addition of fluorine to a protein increases its hydrophobicity while preserving the shape of its side chains, leading to more efficient packing and improved stability.^240,241^ Specifically for enzymes, incorporating fluorine has been noted to improve the activity,^44^ stability,^14,242−246^ specificity,^247,248^ and even shelf life^249^ of each enzyme. Sisila et al. have shown that the global fluorination of cutinase-like enzyme increases its activity at 30 °C by 22% and improves its chemical and thermal stabilities.^44^ Here, the replacement of tyrosine residues with *m*-fluoro-(dl)-tyrosine provides a better substrate binding affinity and enables it to catalyze the synthesis of starch oleate with a degree of substitution (DS) in 24 h of 0.3 ± 0.001 compared to 0.251 ± 0.002 by the wild type; the DS denotes the number of hydroxyl groups substituted per anhydroglucose unit of starch.^250^ Deepankumar et al. have also demonstrated that the replacement of proline residues in transaminase with (4*R*)-fluoroproline confers enhanced thermal stability and higher activity in organic solvents.^40^ Recently, the Tirrell group also demonstrated that the replacement of proline residues in insulin lispro with (4*S*)-fluoroproline improves its stability by delaying the formation of undesired insulin fibrils.^251^ Tobola and colleagues have found that the global fluorination with 5-fluorotryptophan (5FW) increases the melting temperature of *Ralstonia solanacearum* lectin (a carbohydrate-binding protein) and changes its affinity for different sugars.^247^

The effect of fluorination on proteins, known as the “fluorous effect”, causes them to depart from the hydrophilic nature of typical proteins and exhibit self-segregating properties.^42,252,253^ Lee and colleagues emphasize this property by global incorporation of (2*S*,4*R*)-4-fluoroprolines in proline auxotrophic *E. coli* cells to generate a fluorinated target single-chain variable fragment (scFv).^254^ The fluorinated scFv provides an increase in stability at 40 °C and an improved half-life over the wild-type scFv. Tang et al. demonstrate that the fluorination of the hydrophobic core of a coiled-coil protein improves its thermal and chemical stabilities through the incorporation of TFL into a leucine zipper protein.^255^ Similarly, the Montclare group has explored the effects of TFL incorporation on coiled coils, showing large enhancements in thermostability and increased binding of small molecules like curcumin.^246^

While the impact of TFL on protein thermostability has been previously established by Montclare and Tirell,^256^ incorporation of TFL in chloramphenicol acetyltransferase (CAT) results in fluorinated CAT (CAT T), which leads to a 20-fold decrease in activity half-life (*t*_1/2_) compared to wild-type CAT.^256^ However, upon two rounds of random mutagenesis of CAT T in 60 °C activity screenings, a variant with a 27-fold improvement in *t*_1/2_ is generated, demonstrating successful evolution of protein bearing ncAAs. Yoo et al. have had similar success with the evolution of GFP after global incorporation of TFL. Global incorporation of TFL in GFP causes misfolding and loss of fluorescence emission. Through rounds of random mutagenesis and screening via fluorescence-activated cell sorting, a variant with proper folding and nearly identical physical and spectroscopic behavior to that of wild-type GFP was produced.^257^

The Montclare group has further explored both thermostability and supramolecular assembly using elastin-like polypeptide (ELP) block copolymers.^258^ Here, the incorporation of *p*-fluorophenylalanine (pFF) has a notable influence on hydrogel properties observed through rheological characterization. pFF not only affects the secondary structure of the protein, it also allows for the tuning of the transition temperature, *T*_t_. In all ELP block copolymers studied, pFF incorporation results in an increased elastic behavior. Kwon et al. have also studied the hydration dynamics at fluorinated protein surfaces.^259^ Water–protein interactions dictate many processes crucial to protein function including folding, dynamic motions, interactions with other biomolecules, and enzymatic catalysis. By incorporating TFL into a coiled-coil protein and using ultrafast fluorescence spectroscopy, they note that fluorinated side chains exert electrostatic drag on neighboring water molecules, slowing water motion at the protein and potentially causing some changes to its water-mediated interactions.

Finally, the Budisa group has explored routes to high incorporation of fluorine through the use of polyauxotrophic *E. coli* strains for the simultaneous incorporation of multiple ncAAs.^76^ Proline, phenylalanine, and tryptophan have been substituted with fluorinated analogues (4*S*)-fluoroproline, 4-fluorophenylalanine, and 6-fluorotryptophan in *Thermoanaerobacter thermohydrosulfuricus* lipase.^42^ While secondary structure is not substantially affected by ncAA incorporation, the optimal enzyme activity temperature (*T*_opt_) is decreased by about 10 °C, which may be desirable for colder industrial temperatures. The maximum activity, however, is reduced by approximately 60%. Overall, this work establishes a route toward developing functional “Teflon-like” proteins by the integration of multiple monofluorinated ncAAs.^260^

The advantages and disadvantages of SSI and RSI for fluorination applications are similar to those highlighted in the preceding sections. While RSI provides highly efficient methods to generate large signal-to-noise ratio alterations and increases in thermostability, SSI incorporation has been preferred to study specific conformational changes and in ^19^F NMR structural studies. Examples of this have included the site-specific replacement of tyrosine with 2-amino-3-(4-(trifluoromethoxy) phenyl) propanoic acid (OCF_3_Phe) to study a pharmaceutical protein with no adverse impacts on ligand binding and structure and allowing sensitive measurements of the local environments and protein conformational changes.^261^ Another example includes the SSI of ^15^N/^19^F-trifluoromethyl-phenylalanine (^15^N/^19^F-tfmF) in the SHS domain of human vinexin, which allows for detection of P868 ligand binding.^262^

While RSI incorporation does permit similar specific structural dynamics to be detected, as demonstrated in the Tirrell group’s work on the incorporation of proline residues into insulin,^251,263^ its impact on multiple local environments must be considered. As of now, RSI benefits from enabling the generation of highly fluorinated biomolecules that have increased impacts on function and thermostability.

A summary of the fluorinated amino acids and their effects are shown in Table 2.

**Table 2:** Summary of Fluorinated Amino Acids Incorporated through RSI

No.	Peptide material	Fluorinated ncAA incorporated	Function/effect	Ref
1	ω-transaminase	(4*R*)-fluoroproline	Enhances thermal stability and produced higher activity in organic solvents	(40)
2	*T. thermohydrosulfuricus* lipase	(4*S*)-fluoroproline, 4-fluorophenylalanine, 6-fluorotryptophan	Decreases the optimal enzyme activity temperature by 10 °C	(42)
3	cutinase-like enzymes	*m*-fluoro-(dl)-tyrosine	Produces better substrate binding affinity and a higher degree of substitution for the synthesis of starch oleate compared to the wild type	(44)
4	thermoresponsive assembled protein (TRAP)	5,5,5-trifluoroleucine	Produces ^19^F NMR and ^19^F MRS detectable materials	(228)
5	cold shock protein B from *Bacillus subtilis* (BsCspB)	2-fluorophenylalanine, 3-fluorophenylalanine, 4-fluorophenylalanine, 4-fluorotryptophan, 5-fluorotryptophan, 6-fluorotryptophan	Allows for the thermal and structural characterization of the protein using ^19^F NMR and fluorescence spectroscopy	(234, 264)
6	tritrpticin	fluorophenyalanine (*o*, *m* and *p*), 2-fluorotyrosine, 3-fluorotyrosine	Produces ^19^F NMR detectable materials to determine the role of aromatic residues in the antimicrobial activity of the peptide	(235)
7	coiled-coil peptides derived from GCN4	5,5,5-trifluoroisoleucine, (2*S*,3*R*)-4,4,4-trifluorovaline (4TFV)	Improves thermal and chemical stabilities	(243)
8	ω-transaminase	3-fluorotyrosine	Enhances thermostability and organic solvent tolerance without altering substrate specificity and enantioselectivity	(244)
9	*Ralstonia solanacearum* lectin	4-fluorotryptophan, 5-fluorotryptophan, 6-fluorotryptophan, 7-fluorotryptophan	Increases stability and produces greater affinity for specific sugars depending on the residue	(247)
10	p300/CBP associated factor (PCAF)	*o*-fluorophenylalanine (oFF), *m*-fluorophenylalanine (mFF), *p*-fluorophenylalanine (pFF)	pFF leads to improved activity for p53p19 substrate and loss in activity for H3P19 substrate; mFF abolishes the activity for p53P19, while maintaining activity for H3P19; oFF is inactive for both substrates	(248)
11	*Candida antarctica* lipase B, CalB N74D	5-fluoro-L-tryptophan, *m*-fluoro-(dl)-tyrosine, *p*-fluoro-l-phenylalanine	Prolongs the shelf life of the lipase activity	(249)
12	insulin lispro	(4*S*)-fluoroproline, (4*R*)-fluoroproline, 4,4-difluoroproline	(4*S*)-fluoroproline improves insulin lispro stability by delaying insulin fibril formation	(251)
13	single-chain variable fragment (scFv)	(2*S*,4*R*)-4-fluoroprolines	Improves stability and half-life	(254)
14	coiled-coil protein (leucine zipper)	5,5,5-trifluoroleucine	Improves protein resistance to chemical and thermal denaturation	(255)
15	coiled-coil protein fibers	5,5,5-trifluoroleucine	Enhances fiber assembly at pH 8.0 producing thicker and more stable fibers; increases resistance to thermal denaturation	(246)
16	chloramphenicol acetyltransferase (CAT)	5,5,5-trifluoroisoleucine	Produces 27-fold improvement in activity half-life	(256)
17	green fluorescent protein (GFP)	5,5,5-trifluoroleucine	Random mutagenesis and screening via fluorescence-activated cell sorting yielded a mutant similar to wild-type GFP	(257)
18	block copolymer proteins with elastin and COMPcc domains	*p*-fluorophenylalanine	Produces greater elastic character in the materials	(258)
19	coiled-coil protein	5,5,5-trifluoroleucine	Used to study the hydration dynamics at fluorinated surfaces	(259)

### 4.4 Enzyme Engineering

Enzymes have always been very attractive catalysts for industrial processes as biocatalysis does not require the harsh conditions that chemical synthesis does.^7^ The high enantio- and regioselectivities enzymes exhibit toward their substrates and their ability to function in relatively mild conditions are advantageous, improving conversion yields and reducing energy costs.^265,266^ Their main drawback is their inability to withstand the high temperatures and organic solvents required for some production processes, although different approaches such as immobilization and mining enzyme variants from extremophiles (organisms living in extreme conditions) are being used to tackle this problem.^267−269^ Still, it is not an exaggeration to say enzymes are the most utilized class of proteins.^5^

Accordingly, ncAA incorporation into enzymes to improve their activity, selectivity, and chemical stability is well studied.^13,14,270−272^ Pagar and colleagues have a recent review that extensively covers recent advances in biocatalysis, including a comprehensive section on the incorporation of ncAAs and their impact on the functional properties of enzymes.^273^ Additionally, a recent review from Drienovská and colleagues discusses the enhancements in thermal stability achieved through ncAA incorporation.^14^

The global replacement of a cAA with a more hydrophobic ncAA has been shown to improve enzyme activity.^21^ This is seen in the replacement of methionine with the more hydrophobic norleucine, which has long been shown to improve enzyme activity though with varying effects on stability.^274−276^ Cirino et al. observe a nearly 2-fold increase in peroxygenase activity on the incorporation of norleucine into cytochrome P450 BM-3, a fatty acid hydroxylase.^47^ Haernvall et al. also observe a large increase in enzyme activity when they incorporate norleucine into *T. thermohydrosulfuricus* lipase (TTL).^277^ A 50% increase in activity toward ionic phthalic polyesters containing different ether diols is observed in comparison to the parent enzyme without any significant drop in stability.

RSI is less popular for this application as the global incorporation of an ncAA could cause large perturbations in the folded structure of the enzyme and result in the removal of required functional groups, resulting in a loss of activity.^273,278,279^ A more directed SSI might be better for engineering the specific function of the enzyme; however, RSI is still employed and, as demonstrated, often yields positive results for enzyme activity and stability (section 4.3).

## 5 Conclusion

RSI is a critical tool for generating proteins with functions unsupplied by the native amino acid library. Since its inception, it has found numerous applications, which we summarize in this review. In particular, fluorination and labeling techniques have benefited greatly from RSI, owing to the synthesis of ncAAs with fluorinated, alkyne, or azide moieties. Moreover, global ncAA incorporation can also be used to modulate enzymatic activity and protein thermostability.

While a targeted, site-specific approach has several advantages, many of which have been highlighted, RSI offers the ability for facile incorporation of many sites, stemming from its feasibility and cost-effectiveness. Thus, RSI has been popular in generating large changes to protein thermostability as well as increased signal-to-noise ratios in NMR signaling and ncAA-based labeling.

It is expected that RSI will bridge the gap to industrial scale processes with the development of methods that allow for the simultaneous incorporation of multiple ncAAs or with new techniques such as THRONCAT, which utilizes RSI without the use of auxotrophic cell lines. Further research is required to scale up protein production with RSI to an industrial level. Streamlining the process and developing new ncAAs with greater affinity to native biological machinery will be crucial for maximizing incorporation efficiency and expanding the application of this technique.
